# Analysis of the clinical value of anterior peritoneal reflection for the management of rectal cancer

**DOI:** 10.1093/gastro/goaf064

**Published:** 2025-07-16

**Authors:** Huaqing Zhang, Guole Lin, Bin Wu, Huizhong Qiu, Junyang Lu, Xiyu Sun, Beizhan Niu, Lai Xu, Guannan Zhang, Zhen Sun, Kexuan Li, Yi Xiao

**Affiliations:** Division of Colorectal Surgery, Department of General Surgery, Peking Union Medical College Hospital, Chinese Academy of Medical Science & Peking Union Medical College, Beijing, P. R. China; Division of Colorectal Surgery, Department of General Surgery, Peking Union Medical College Hospital, Chinese Academy of Medical Science & Peking Union Medical College, Beijing, P. R. China; Division of Colorectal Surgery, Department of General Surgery, Peking Union Medical College Hospital, Chinese Academy of Medical Science & Peking Union Medical College, Beijing, P. R. China; Division of Colorectal Surgery, Department of General Surgery, Peking Union Medical College Hospital, Chinese Academy of Medical Science & Peking Union Medical College, Beijing, P. R. China; Division of Colorectal Surgery, Department of General Surgery, Peking Union Medical College Hospital, Chinese Academy of Medical Science & Peking Union Medical College, Beijing, P. R. China; Division of Colorectal Surgery, Department of General Surgery, Peking Union Medical College Hospital, Chinese Academy of Medical Science & Peking Union Medical College, Beijing, P. R. China; Division of Colorectal Surgery, Department of General Surgery, Peking Union Medical College Hospital, Chinese Academy of Medical Science & Peking Union Medical College, Beijing, P. R. China; Division of Colorectal Surgery, Department of General Surgery, Peking Union Medical College Hospital, Chinese Academy of Medical Science & Peking Union Medical College, Beijing, P. R. China; Division of Colorectal Surgery, Department of General Surgery, Peking Union Medical College Hospital, Chinese Academy of Medical Science & Peking Union Medical College, Beijing, P. R. China; Division of Colorectal Surgery, Department of General Surgery, Peking Union Medical College Hospital, Chinese Academy of Medical Science & Peking Union Medical College, Beijing, P. R. China; Division of Colorectal Surgery, Department of General Surgery, Peking Union Medical College Hospital, Chinese Academy of Medical Science & Peking Union Medical College, Beijing, P. R. China; Division of Colorectal Surgery, Department of General Surgery, Peking Union Medical College Hospital, Chinese Academy of Medical Science & Peking Union Medical College, Beijing, P. R. China

**Keywords:** rectal neoplasms, anterior peritoneal reflection, neoadjuvant chemoradiotherapy, survival analysis, lateral lymph node, metastases

## Abstract

**Background:**

Tumor location affects rectal cancer management, but no consensus exists on criteria. The anterior peritoneal reflection (aPR), an anatomical landmark, shows potential for defining tumor location but requires clinical validation. This study evaluated the utility of aPR in guiding neoadjuvant chemoradiotherapy (nCRT) decisions and predicting lateral lymph node (LLN)/distant metastasis patterns.

**Methods:**

This single-center retrospective cohort analyzed data from Peking Union Medical College Hospital (Beijing, China) between January 2016 and August 2022. Magnetic resonance imaging (MRI)-measured aPR parameters were pathologically validated. Patients were stratified by aPR-based definition and tumor height (10 cm). Kaplan–Meier survival curves, log-rank tests, and Cox regression were used for prognostic analysis.

**Results:**

Among 588 patients (439 tumors ≥5 cm from the anal verge), MRI identified aPR with an accuracy of 95.4%. For tumors ≥5 cm, aPR-defined middle-to-low rectal cancer showed lower 3-year disease-free survival (DFS) rate than the upper rectal cancer (*P *= 0.010), while their 3-year overall survival (OS) rates were comparable. Conversely, 10-cm-defined classification showed no DFS or OS differences (both *P *> 0.2). Cox regression confirmed aPR-defined classification as an independent DFS predictor (HR = 3.19, *P *= 0.014), while 10-cm classification was non-predictive. nCRT with tumor regression grade (TRG) 0–1 trended toward improved DFS compared with direct surgery (HR = 0.56, *P *= 0.072). The independent protective effect of nCRT with TRG 0–1 for DFS was exclusive to the aPR-defined middle-to-low rectal cancer subgroup (HR = 0.45, *P *= 0.026) and not observed in the 10-cm subgroup. aPR-defined classification was independently associated with LLNs on MRI and postoperative pulmonary metastasis.

**Conclusion:**

aPR may guide nCRT decision-making and predict LLN metastasis and postoperative distant organ metastasis.

## Introduction

Rectal cancer accounts for more than 50% of colorectal cancer cases, with middle-to-low rectal cancers constituting 60% to 75% of the total incidence of rectal cancer [1]. Tumor location significantly affects the prognosis of rectal cancer, and directly informs treatment strategies. Due to their proximity to surrounding pelvic organs and complexity of the anal structure, along with the presence of lateral lymphatic drainage pathways and the inherent challenges of achieving R0 resection, middle-to-low locally advanced rectal cancer (LARC) differs markedly from upper rectal cancer in terms of the indication of neoadjuvant chemoradiotherapy (nCRT), lateral lymph node (LLN) metastasis, and postoperative distant organ metastatic patterns [2]. Current definitions of rectal segments lack consensus. Some guidelines define rectal segments based on a specific distance from the anal verge [3, 4]. For instance, the Chinese Society for Clinical Oncology (CSCO) guideline classifies middle-to-low rectal cancer as a tumor of which the lower edge locates within 10 cm of the anal verge on magnetic resonance imaging (MRI), while others adopt the anterior peritoneal reflection (aPR) as a classification standard [5]. Considering the heterogeneity in prognosis and treatment among patients defined by various methods [6], identifying optimal segmentation criteria for rectal cancer is crucial.

Compared with other methods, a PR-defined classification provides a clearer anatomical distinction, as the rectum located above or below the aPR differs in the embryological, anatomical, and lymphatic characteristics [7]. Despite these advantages, only one study to date has reported the application of aPR in guiding postoperative radiotherapy decisions for rectal cancer [6], highlighting the necessity for further validation.

This study aimed to assess the clinical utility of the aPR in the management of rectal cancer. The primary focus was to determine whether aPR can accurately identify patients who might benefit from nCRT. Additionally, the study investigated whether employing aPR for tumor localization enhances the prediction of LLN involvement and the patterns of distant metastasis.

## Patients and methods

### Patients

This retrospective, single-center observational study utilized clinicopathological and follow-up data from the prospective rectal cancer database of the Colorectal Surgery Division at Peking Union Medical College Hospital (PUMCH; Beijing, China), covering the period from January 2016 to August 2022. This study was approved by the Ethics Committee of PUMCH, which waived the requirement for informed consent as it was a retrospective study. The study was conducted in accordance with the principles of the Declaration of Helsinki.

Previous research from our center reported an average aPR height of 98.7 ± 14.4 mm (range, 63.6–154 mm) in Chinese rectal cancer patients [8]. Consequently, tumors with a distal margin ≤5 cm from the anal verge were clearly classified as middle-to-low rectal cancers, whereas those located 5–15 cm were considered “middle-to-upper” rectal cancers, a category with potential definitional conflicts. Therefore, only patients with tumors 5–15 cm from the anal verge were included in the analysis evaluating nCRT benefits, while all patients were included in the remaining analyses.

Given the limited literature comparing different definitions of tumor locations in rectal cancer, accurately predicting the required sample size was challenging. Nevertheless, the study’s primary methodology was multivariable Cox proportional hazards regression analysis, emphasizing disease-free survival (DFS). Assuming a DFS event rate of 20%–25% in LARC patients [9], we anticipated incorporating up to 10 independent variables into the final multivariable Cox model. According to the Events per Variable principle [10], the number of events should be at least 10 times the number of independent variables. Hence, a minimum sample size of 400 were considered necessary.

### Selection criteria

The patients were included in this study if they met the following criteria. (i) Adult patients (≥18 years) with pathologically confirmed rectal adenocarcinoma; (ii) Tumor within 15 cm from the anal verge by MRI; (iii) LARC: for those receiving nCRT, baseline MRI indicated clinical staging of T3/4 or N+; for those undergoing direct surgery, postoperative pathological stage was T3/4 or N+; and (iv) Underwent radical surgical resection.

Exclusion criteria were as follows: (i) Multiple primary colorectal cancers; (ii) Distant metastases before surgery (M1); (iii) History of previous malignant tumors; (iv) Positive resection margin (including proximal, distal and circumferential); (v) Emergency surgery required due to intestinal obstruction or perforation; or (vi) Inability to identify aPR on MRI.

### Observational parameters

The following information of all patients was collected. (i) General indicators: sex, age, body mass index (BMI), and American Society of Anesthesiologists (ASA) stage; (ii) Tumor characteristics: baseline MRI-assessed T stage (mrT), MRI-assessed N stage (mrN), distance from the lower edge of the tumor to the anal verge, mesorectal fascia (MRF), and extramural vascular invasion (EMVI); (iii) Pathological features and postoperative treatment: tumor pathological stage, differentiation, perineural invasion, lymphovascular invasion, tumor regression grade (TRG), and details of adjuvant therapy; (iv) Postoperative survival: DFS and overall survival (OS).

### Definition of parameters

Tumor location was defined by using both the aPR method and the measured distance between the tumor’s distal margin and the anal verge. Tumors that straddled or extended below the aPR, and those located ≤10 cm from the anal verge on MRI, were categorized as “middle-to-low rectal cancer” under their respective definitions. Tumor direction was evaluated on axial T2-weighted MRI (T2WI) and categorized as “anterior,” “posterior,” “lateral,” or “circumferential.” Tumor pathological staging adhered to the eighth edition of the American Joint Committee on Cancer (AJCC) Tumor, Node, Metastasis (TNM) staging system [11]. TRG followed the standards of the American Pathologists Association [12]: Grade 0 (no tumor cells, complete response), Grade 1 (rare residual cancer cells, near complete response), Grade 2 (residual tumor with regression, partial response), and Grade 3 (minimal or no regression). In this study, TRG was recorded as a three-category variable: TRG 0–1, TRG 2, and TRG 3. DFS was defined as the time from surgery to the first local recurrence, distant metastasis, or death from any cause. OS was defined as the duration from surgery to death from any cause. Distant organ metastasis was defined as metastases detected in organs such as the liver, lungs, or bones by using imaging modalities (e.g. computed tomography [CT], MRI, radionuclide scans), without the need for pathological confirmation.

### Measurements

**Figure 1.**
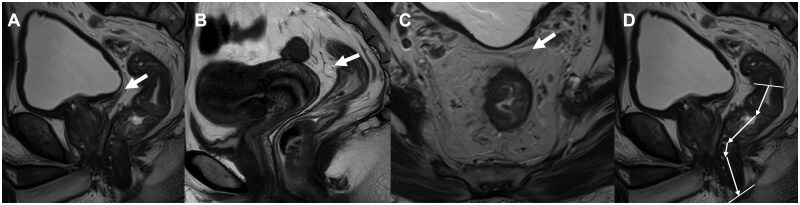
Identification of the anterior peritoneal reflection (aPR) and measurement of aPR height on magnetic resonance imaging. (A) aPR (arrow) in the sagittal plane (male). (B) aPR (arrow) in the sagittal plane (female). (C) The “seagull sign” (arrow) of aPR in the axial plane. (D) The measurement of aPR height in the sagittal plane of magnetic resonance imaging

Identification and measurement were performed on sagittal and axial T2WI images by radiologists with at least 5 years of specialized experience using aPR-related parameters. The aPR was visualized as a low-signal line at the apex of the seminal vesicles (in males) or at the cervical angle (in females) [6] (Figure 1A and B). Axial images aided in identifying the aPR, characterized by the “seagull sign” [13] (Figure 1C). On sagittal images, lines were drawn from the anal verge to the aPR along the rectal centerline, and their total length was recorded as the aPR height (Figure 1D). By using the same method, distances from the tumor’s lower edge to both the anal verge (distance to the anal verge) and the aPR (distance to the aPR) were measured. If the tumor was located straddling or below the aPR, the distance to the aPR was recorded as a positive value; otherwise, it was recorded as negative. Drawing on the classification of esophagogastric junction tumors (Siewert classification) [14], we categorized the distance to the aPR into a trichotomous variable: ≥5 cm, 0–5 cm and <0 cm. All measurements were performed twice, and the averages were used for subsequent analyses. Additionally, aPR-related parameters were reviewed and measured immediately on surgical specimens and documented in the formatted operative reports. Subsequent statistical analyses were based on the findings from surgical specimens.

Based on MRI, LLNs were evaluated in the internal iliac, obturator, external iliac, and common iliac regions on baseline axial T2WI and diffusion-weighted MRI images. The diagnosis of clinical LLN metastasis includes (i) the short axis of LLN ≥7 mm; and (ii) the short axis of LLN <7 mm but exhibiting two or more characteristics of malignancy (irregular borders, heterogeneous signal intensity, and round shape) [15]. Typical benign lymph nodes (clear borders, flat shape, uniform signal intensity) were not recorded [16].

### nCRT, surgery, adjuvant therapy and follow-up

nCRT was routinely recommended for patients with baseline MRI indicating LARC. At our center, there was no strict uniform criterion regarding tumor distance for nCRT eligibility. Most practices adhered to the CSCO guidelines, recommending nCRT for tumors ≤10 cm from the anal verge. However, some patients declined nCRT and opted for direct surgery instead.

Surgery was performed 8–10 weeks after completing radiotherapy, with the specific surgical approach determined based on the patient’s general condition, tumor location, and staging. All surgeries were performed by surgeons with at least 100 colorectal cancer operations annually, adhering to a standardized total mesorectal excision (TME) protocol.

Adjuvant chemotherapy commenced 4–8 weeks post-surgery, including XELOX, mFOLFOX6, or single-agent capecitabine. Patients who underwent nCRT were recommended to receive adjuvant chemotherapy regardless of the pathological stage. For patients who had direct surgery, postoperative CRT was recommended. However, some patients refused or could not undergo postoperative treatment due to frailty.

After routine discharge, patients returned for outpatient follow-up two weeks post-surgery, with further treatments guided by postoperative pathology and recovery. Follow-ups were subsequently conducted by research assistants. Patients were advised to undergo follow-up examinations every 3 to 4 months during the first 2 years postoperatively, every 6 months from 2 to 5 years, and annually thereafter. Follow-up evaluations included tumor marker tests, abdominal ultrasound, chest X-ray, annual enhanced CT scans of the chest, abdomen, and pelvis, and colonoscopy. Local recurrence and distant metastasis were confirmed by biopsy when appropriate, or by progressive lesion enlargement or the appearance of new lesions.

### Statistical analysis

Statistical analyses were conducted by using SPSS version 26.0 and R version 4.1.2. The Shapiro–Wilk test was employed to assess the normality of continuous variables. Normally distributed data are presented as mean ± standard deviation and were compared by using the *t*-test. Skewed data are presented as median (interquartile range) and were compared by using non-parametric test. Categorical data are presented as frequency (percentage) and were compared by using the Chi-square test or Fisher’s exact test. Differences in survival rates between groups were analyzed by using the Kaplan-–Meier method and log-rank test. Cox proportional hazards regression models were utilized to identify factors affecting survival rates, with subgroup analyses performed accordingly. Variables with a *P* value of ≤0.2 in univariate regression models were included in the multivariate analysis, which employed forward stepwise regression. A *P* value of <0.05 was considered statistically significant. The R packages used in the analysis included “tableone,” “survey,” “readxl,” “epicalc,” “survival,” “survminer,” and “forestploter.”

## Results

### Patient characteristics

**Figure 2.**
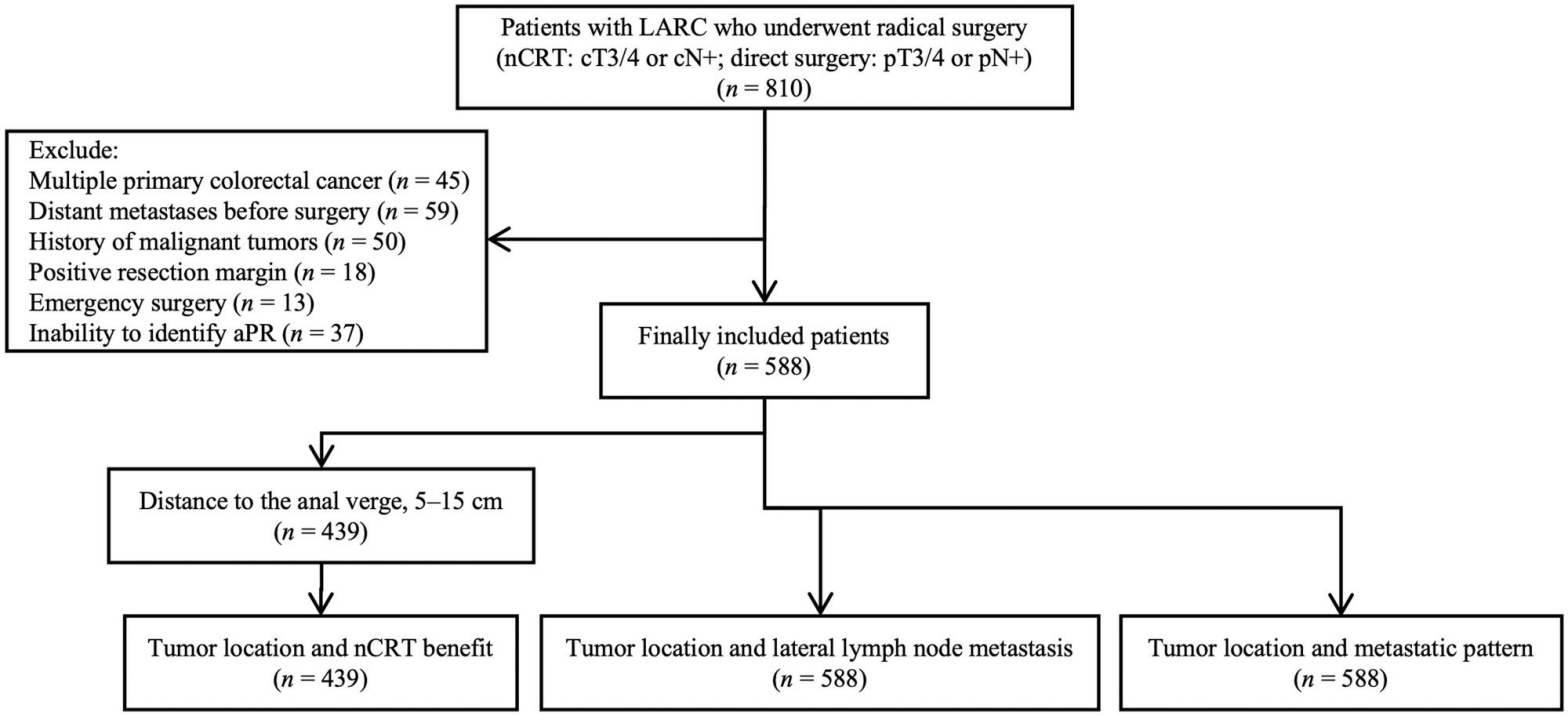
Flow chart of patient selection.

A total of 588 patients were included in this study, with 62 having tumors above the aPR and 526 with tumors straddling or below it. Among these, 439 patients had tumors located ≥5 cm from the anal verge. The flowchart of patient selection is shown in Figure 2. The median follow-up period was 40 months (interquartile range, 29 to 63 months). Demographic, clinicopathological, and treatment data are presented in Table 1. The MRI accuracy in identifying tumors located above or straddling/below the aPR was 91.9% and 95.8%, respectively. The details are shown in Table 2.

**Table 1.** The demographic, clinicopathological and treatment details of the study patients (Statistically significant *P*-values with a level of significance set at <0.05 are highlighted in bold).

Variable	Tumor location relative to the aPR			Tumor location relative to the anal verge		
	Straddle or below (*n *= 526)	Above (*n *= 62)	*P* value	≤10 cm (*n *= 518)	>10 cm (*n *= 70)	*P* value
Sex, *n* (%)			**0.038**			0.360
Female	153 (29.1)	26 (41.9)		161 (31.1)	18 (25.7)	
Male	373 (70.9)	36 (58.1)		357 (68.9)	52 (74.3)	
Age, *n* (%)			0.291			0.216
<45	51 (9.7)	10 (16.1)		50 (9.7)	11 (15.7)	
45–65	273 (51.9)	30 (48.4)		272 (52.5)	31 (44.3)	
≥65	202 (38.4)	22 (35.5)		196 (37.8)	28 (40.0)	
BMI, *n* (%)			0.713			0.803
<24 kg/m^2^	242 (46.0)	27 (43.5)		236 (45.6)	33 (47.1)	
≥24 kg/m^2^	284 (54.0)	35 (56.5)		282 (54.4)	37 (52.9)	
ASA stage, *n* (%)			0.131			0.609
I	121 (23.0)	20 (32.3)		121 (23.4)	20 (28.6)	
II	371 (70.5)	36 (58.1)		362 (69.88)	45 (64.3)	
III	34 (6.5)	6 (9.6)		35 (6.8)	5 (7.1)	
mrT, *n* (%)			0.357			**<0.001**
T2	31 (5.9)	1 (1.6)		31 (6.0)	1 (1.4)	
T3	405 (77.0)	49 (79.0)		408 (78.8)	46 (65.7)	
T4	90 (17.1)	12 (19.4)		79 (15.2)	23 (32.9)	
mrN, *n* (%)			0.782			0.964
N0	28 (5.3)	4 (6.5)		28 (5.4)	4 (5.7)	
N1	186 (35.4)	24 (38.7)		186 (35.9)	24 (34.3)	
N2	312 (59.3)	34 (54.8)		304 (58.7)	42 (60.0)	
MRF positive, *n* (%)	169 (32.1)	17 (27.4)	0.451	160 (30.9)	26 (37.1)	0.291
EMVI positive, *n* (%)	212 (40.3)	24 (38.7)	0.809	201 (38.8)	35 (50.0)	0.073
Tumor direction, *n* (%)			0.078			0.132
Anterior	133 (25.3)	13 (21.0)		128 (24.7)	18 (25.7)	
Posterior	127 (24.1)	24 (38.7)		128 (24.7)	23 (32.9)	
Lateral	157 (29.9)	17 (27.4)		152 (29.3)	22 (31.4)	
Circumferential	109 (20.7)	8 (12.9)		110 (21.3)	7 (10.0)	
Distance to the anal verge, cm	6.3 (4.7, 7.7)	11.0 (9.9, 12.2)	**<0.001**	6.2 (4.7, 7.6)	11.1 (10.5, 12.4)	**<0.001**
Distance to the aPR, cm	3.5 (2.0, 5.0)	−1.2 (−2.3, −0.5)	**<0.001**	3.5 (2.1, 5.1)	−0.9 (−2.2, 0.6)	**<0.001**
mrLLN present, *n* (%)	254 (48.3)	7 (11.3)	**<0.001**	254 (49.0)	7 (10.0)	**<0.001**
Treatment and response, *n* (%)			**<0.001**			**<0.001**
Direct surgery	121 (23.0)	44 (71.0)		128 (24.7)	37 (52.9)	
nCRT (TRG 0–1)	211 (40.1)	7 (11.3)		206 (39.8)	12 (17.1)	
nCRT (TRG 2)	173 (32.9)	10 (16.1)		165 (31.8)	18 (25.7)	
nCRT (TRG 3)	21 (3.9)	1 (1.6)		19 (3.7)	3 (4.2)	
pTNM stage, *n* (%)			**<0.001**			**<0.001**
pCR	105 (20.0)	5 (8.1)		103 (19.9)	7 (10.0)	
I	115 (21.9)	2 (3.2)		113 (21.8)	4 (5.7)	
II	157 (29.9)	31 (50.0)		157 (30.3)	31 (44.2)	
III	149 (28.2)	24 (38.7)		145 (28.0)	28 (40.0)	
Well differentiation, *n* (%)	497 (94.5)	57 (91.9)	0.599	490 (94.6)	64 (91.4)	0.428
Tumor deposit positive, *n* (%)	37 (7.0)	12 (19.3)	**<0.001**	38 (7.3)	11 (15.7)	**0.017**
Lymphovascular invasion positive, *n* (%)	63 (12.0)	11 (17.7)	0.196	58 (11.2)	16 (22.9)	**0.006**
Perineural invasion positive, *n* (%)	47 (8.9)	11 (17.7)	**0.028**	46 (8.9)	12 (17.1)	**0.030**
Postoperative therapy, *n* (%)	396 (75.3)	45 (72.6)	0.642	388 (74.9)	53 (75.7)	0.883

aPR, anterior peritoneal reflection; ASA, American Society of Anesthesiologists; BMI, body mass index; EMVI, extramural vascular invasion; MRF, mesorectal fascia; mrLLN, magnetic resonance imaging-assessed lateral lymph node; mrN, magnetic resonance imaging-assessed N stage; mrT, magnetic resonance imaging-assessed T stage; nCRT, neoadjuvant chemoradiotherapy; pCR, pathological complete response.

**Table 2.** Tumor location relative to the aPR as determined by MRI and surgical findings.

aPR relationship	By surgical findings	Above (*n *= 62)	Straddle/below (*n *= 526)	Overall accuracy
By MRI	Above	57 (91.9%)	22 (4.2%)	
	Straddle/below	5 (8.1%)	504 (95.8%)	
Accuracy rate		91.9%	95.8%	95.4%

aPR, anterior peritoneal reflection; MRI, magnetic resonance imaging.

### aPR and nCRT benefit

**Figure 3.**
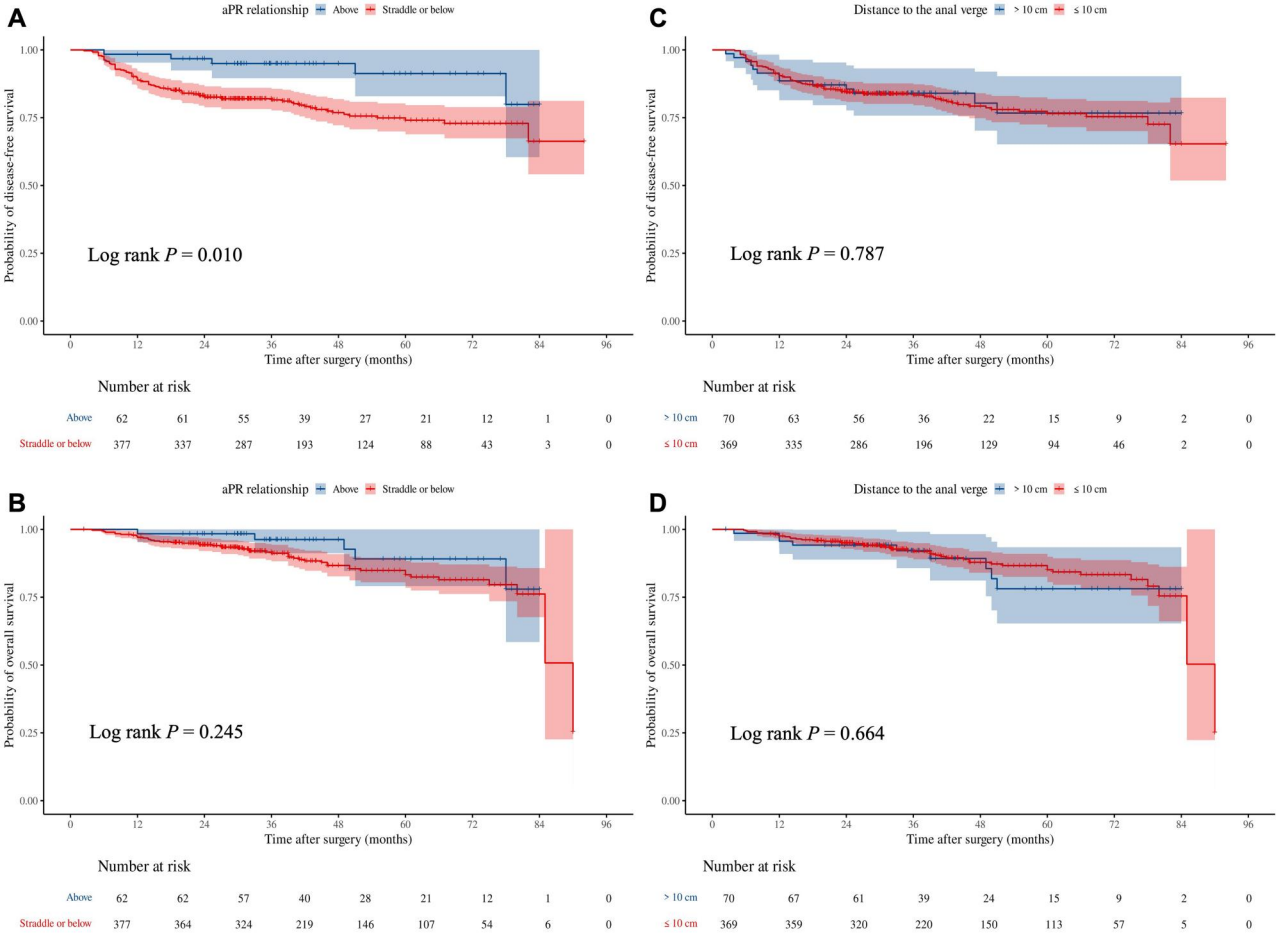
Comparison of prognostic differences in rectal cancer based on various tumor location definitions. (A) The Kaplan-Meier curves of disease-free survival between tumors straddling/below and above the aPR. (B) The Kaplan-Meier curves of overall survival between tumors straddling/below and above the aPR. (C) The Kaplan-Meier curves of disease-free survival between tumors >10 cm and ≤10 cm from the anal verge. (D) The Kaplan-Meier curves of overall survival between tumors >10 cm and ≤10 cm from the anal verge.

For the 439 patients with tumors located ≥5 cm from the anal verge, 55 patients (12.5%) died and 74 patients (16.9%) experienced tumor progression during follow-up. The 3-year DFS and OS rates for patients with tumors straddling or below the aPR were 81.6% and 91.2%, respectively. In contrast, patients with tumors above the aPR had a 3-year DFS rate of 95.0% and a 3-year OS rate of 96.2%. The 3-year DFS rate for tumors straddling or below the aPR was significantly lower than that for tumors above the aPR (*P *= 0.010, Figure 3A), while there was no statistically significant difference in OS between the two groups (*P *= 0.245, Figure 3B). For patients with tumors ≤10 cm from the anal verge, the 3-year DFS rate was 83.4% and the 3-year OS rate was 91.9%, compared with a 3-year DFS rate of 84.0% and a 3-year OS rate of 92.2% for patients with tumors >10 cm from the anal verge. There was no statistically significant difference in either DFS or OS between these two distance-based groups (*P *= 0.787 and 0.664, respectively; Figure 3C and D).

The Cox proportional hazards model showed that the tumor relationship to the aPR was an independent prognostic factor for DFS (HR, 3.19; 95% CI, 1.26–8.06; *P *= 0.014), while the distance from the anal verge was not. Regarding treatment and response, nCRT with TRG 0–1 showed a trend toward improved DFS compared with direct surgery (HR, 0.56; 95% CI, 0.29–1.06; *P *= 0.072), while nCRT with TRG 2 or TRG 3 did not significantly influence DFS. The details are shown in Table 3.

**Table 3.** Univariate and multivariate analyses of risk factors of DFS using a Cox regression model for 439 cases of rectal cancer

Variable	Univariate analysis		Multivariate analysis	
	HR (95% CI)	*P* value	HR (95% CI)	*P* value
Treatment and response				
Direct surgery	1.00 (Reference)		1.00 (Reference)	
nCRT (TRG 0–1)	0.67 (0.37–1.23)	0.198	0.56 (0.29–1.06)	0.072
nCRT (TRG 2)	1.71 (1.04–2.81)	**0.034**	1.22 (0.71–2.07)	0.471
nCRT (TRG 3)	1.96 (0.85–4.52)	0.115	1.62 (0.67–3.95)	0.285
Tumor location relative to the aPR (straddle/below vs above)	3.09 (1.25–7.62)	**0.014**	3.19 (1.26–8.06)	**0.014**
Tumor location relative to the anal verge (≤10 cm vs >10 cm)	1.08 (0.60–1.96)	0.787		
Sex (male vs female)	1.06 (0.66–1.70)	0.823		
Age				
<45 years	1.00 (Reference)		1.00 (Reference)	
45–65 years	1.29 (0.51–3.29)	0.588	1.40 (0.54–3.68)	0.491
≥65 years	2.21 (0.87–5.57)	0.094	2.89 (1.09–7.66)	**0.033**
BMI (<24 kg/m^2^ vs ≥24 kg/m^2^)	0.99 (0.65–1.51)	0.961		
ASA stage				
I	1.00 (Reference)		1.00 (Reference)	
II	0.72 (0.46–1.14)	0.165	0.61(0.37–0.99)	**0.044**
III	0.86 (0.33–2.24)	0.755	0.51 (0.19–1.39)	0.188
mrT				
T2	1.00 (Reference)		1.00 (Reference)	
T3	1.30 (0.32–5.33)	0.714	0.80 (0.19–3.39)	0.756
T4	2.90 (0.69–12.21)	0.146	1.52 (0.34–6.83)	0.584
mrN				
N0	1.00 (Reference)			
N1	1.37 (0.42–4.50)	0.607		
N2	1.47 (0.46–4.68)	0.518		
MRF (positive vs negative)	1.31 (0.85–2.03)	0.219		
EMVI (positive vs negative)	2.15 (1.40–3.31)	**<0.001**	1.68 (1.06–2.67)	**0.028**
Tumor direction				
Anterior	1.00 (Reference)			
Posterior	1.30 (0.68–2.49)	0.434		
Lateral	1.34 (0.74–2.41)	0.336		
Circumferential	1.19 (0.58–2.45)	0.639		
mrLLN (positive vs negative)	1.85 (1.17–2.93)	**0.008**	1.63 (1.01–2.64)	**0.047**
Differentiation (poor vs well/moderate)	1.66 (0.80–3.43)	0.174	1.29 (0.58–2.84)	0.531
Tumor deposit (positive vs negative)	0.67 (0.29–1.53)	0.338		
Lymphovascular invasion (positive vs negative)	1.40 (0.82–2.37)	0.216		
Perineural invasion (positive vs negative)	1.63 (0.92–2.90)	0.092	1.14 (0.60–2.14)	0.689
Postoperative therapy (yes vs no)	1.00 (0.62–1.62)	0.998		

Statistically significant *P*-values with a level of significance set at <0.05 are highlighted in bold. ASA, American society of anesthesiologists; BMI, body mass index; CI, confidence interval; EMVI, extramural vascular invasion; HR, hazard ratio; MRF, mesorectal fascia; mrLLN, magnetic resonance imaging-assessed lateral lymph node; mrN, magnetic resonance imaging-assessed N stage; mrT, magnetic resonance imaging-assessed T stage; nCRT, neoadjuvant chemoradiotherapy.

**Figure 4.**
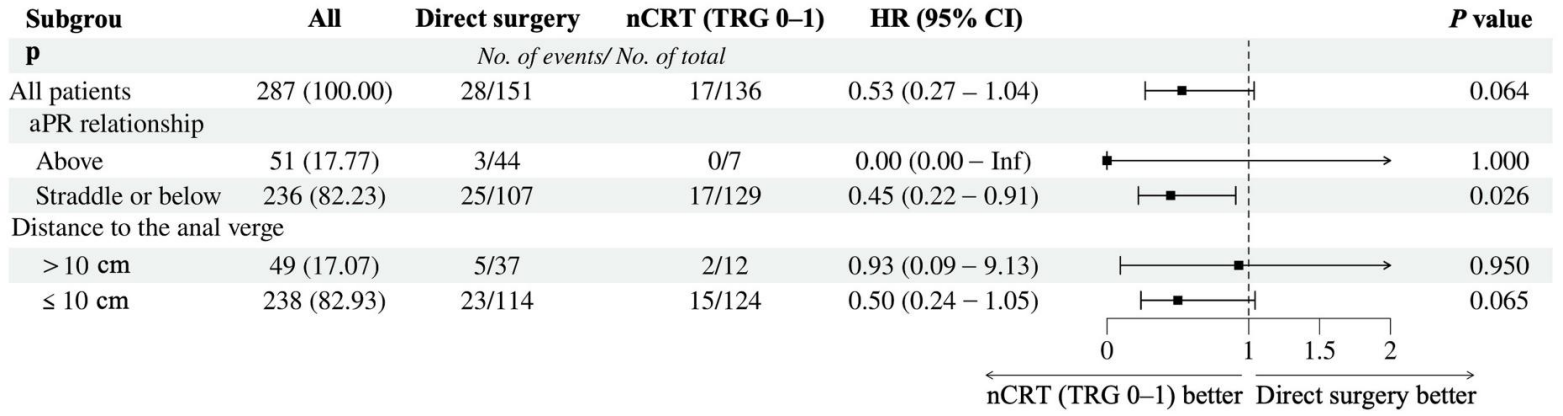
Subgroup analysis of the Cox regression model for disease-free survival. Adjusted for age, ASA stage, EMVI and mrLLN. aPR, anterior peritoneal reflection; ASA, American society of anesthesiologists; EMVI, extramural vascular invasion; mrLLN, magnetic-resonance-imaging-assessed lateral lymph node; nCRT, neoadjuvant chemoradiotherapy; TRG, tumor regression grade.

Subgroup analysis revealed that for patients with cancer straddling or below the aPR, nCRT with TRG 0–1 was an independent protective factor for DFS (HR, 0.45; 95% CI, 0.22–0.91; *P *= 0.026). In contrast, this relationship was not observed when tumor location was defined by the distance from the anal verge. The details are illustrated in Figure 4.

### aPR and lateral lymph node

Binary logistic regression indicated that the distal extension of the tumor away from the aPR was an independent factor associated with the presence of LLNs on MRI (≥5 cm: reference; 0–5 cm: OR, 0.60; 95% CI, 0.36–0.98; *P *= 0.041; <0 cm: OR, 0.23; 95% CI, 0.08–0.64; *P *= 0.005), whereas the distance to the anal verge did not show a consistent, significant association. The details are shown in Table 4.

**Table 4.** Univariate and multivariate analyses of related factors of mrLLN using a binary logistic regression model for 588 cases of rectal cancer

Variable	Univariate analysis		Multivariate analysis	
	OR (95% CI)	*P* value	OR (95% CI)	*P* value
Sex (male vs female)	1.16 (0.81–1.65)	0.422		
Age				
<45 years	1.00 (Reference)		1.00 (Reference)	
45–65 years	0.79 (0.45–1.37)	0.399	0.69 (0.37–1.30)	0.251
≥65 years	0.59 (0.33–1.04)	0.066	0.54 (0.28–1.02)	0.059
BMI (<24 kg/m^2^ vs ≥24 kg/m^2^)	1.30 (0.94–1.80)	0.117	1.36 (0.96–1.94)	0.086
ASA stage				
I	1.00 (Reference)			
II	1.00 (0.68–1.47)	0.994		
III	0.83 (0.40–1.69)	0.599		
mrT				
T2	1.00 (Reference)			
T3	1.54 (0.73–3.28)	0.258		
T4	1.63 (0.71–3.73)	0.246		
mrN				
N0	1.00 (Reference)		1.00 (Reference)	
N1	1.38 (0.62–3.07)	0.427	1.40 (0.60–3.26)	0.434
N2	2.12 (0.98–4.62)	0.057	2.25 (0.98–5.18)	0.056
MRF (positive vs negative)	1.48 (1.05–2.10)	**0.027**	1.40 (0.94–2.07)	0.094
EMVI (positive vs negative)	1.07 (0.77–1.49)	0.704		
Tumor direction				
Anterior	1.00 (Reference)			
Posterior	0.84 (0.53–1.34)	0.469		
Lateral	1.15 (0.74–1.79)	0.528		
Circumferential	0.74 (0.45–1.21)	0.226		
Differentiation (poor vs well/moderate)	0.67 (0.32–1.38)	0.274		
The distance to the anal verge				
≤5 cm	1.00 (Reference)		1.00 (Reference)	
5–10 cm	0.64 (0.44–0.94)	**0.021**	0.77 (0.47–1.24)	0.283
>10 cm	0.08 (0.04–0.20)	**<0.001**	0.15 (0.06–0.41)	**<0.001**
The distance to the aPR				
≥5 cm	1.00 (Reference)		1.00 (Reference)	
0–5 cm	0.51 (0.34–0.76)	**0.001**	0.60 (0.36–0.98)	**0.041**
<0 cm	0.09 (0.04–0.20)	**<0.001**	0.23 (0.08–0.64)	**0.005**

Statistically significant *P*-values with a level of significance set at <0.05 are highlighted in bold. aPR, anterior peritoneal reflection; ASA, American Society of Anesthesiologists; BMI, body mass index; CI, confidence interval; EMVI, extramural vascular invasion; MRF, mesorectal fascia; mrN, magnetic resonance imaging-assessed N stage; mrT, magnetic resonance imaging-assessed T stage; OR, odds ratio.

### aPR and patterns of distant organ metastasis

Among all patients, 57 cases (9.6%) developed pulmonary metastases during follow-up. The Cox proportional hazards regression model for factors influencing pulmonary metastasis after surgery indicated that the distance to the aPR was an independent prognostic factor for pulmonary metastasis (≥5 cm: reference; 0–5 cm: HR, 0.59; 95% CI, 0.38–0.92; *P *= 0.021; <0 cm: HR, 0.20; 95% CI, 0.07–0.54; *P *= 0.001), whereas the distance to the anal verge was not. The details are shown in Table 5.

**Table 5.** Univariate and multivariate analyses of risk factors of pulmonary metastases using a Cox regression model for 588 cases of rectal cancer

Variables	Univariate analysis		Multivariate analysis	
	HR (95% CI)	*P* value	HR (95% CI)	*P* value
Sex (male vs female)	1.15 (0.77–1.72)	0.495		
Age				
<45 years	1.00 (Reference)			
45–65 years	0.89 (0.47–1.72)	0.736		
≥65 years	1.47 (0.77–2.81)	0.238		
BMI (<24 kg/m^2^ vs ≥24 kg/m^2)^	1.02 (0.71–1.47)	0.909		
ASA stage				
I	1.00 (Reference)			
II	0.80 (0.54–1.19)	0.262		
III	0.66 (0.28–1.58)	0.354		
mrT				
T2	1.00 (Reference)		1.00 (Reference)	
T3	2.03 (0.64–6.44)	0.227	1.80 (0.56–5.84)	0.325
T4	4.21 (1.29–13.72)	**0.017**	4.02 (1.14–14.19)	**0.030**
mrN				
N0	1.00 (Reference)			
N1	1.71 (0.61–4.76)	0.305		
N2	1.76 (0.64–4.82)	0.272		
MRF (positive vs negative)	1.43 (0.99–2.08)	0.058	0.78 (0.49–1.24)	0.288
EMVI (positive vs negative)	1.99 (1.39–2.85)	**<0 .001**	1.72 (1.15–2.57)	**0.008**
Tumor direction				
Anterior	1.00 (Reference)			
Posterior	0.91 (0.45–1.84)	0.792		
Lateral	0.83 (0.42–1.63)	0.589		
Circumferential	0.59 (0.25–1.38)	0.226		
mrLLN (positive vs negative)	1.48 (1.03–2.12)	**0.034**	1.42 (0.98–2.08)	0.067
The distance to the anal verge				
≤5 cm	1.00 (Reference)			
5–10 cm	0.94 (0.63–1.42)	0.783		
>10 cm	0.89 (0.47–1.68)	0.710		
The distance to the aPR				
≥5 cm	1.00 (Reference)		1.00 (Reference)	
0–5 cm	0.86 (0.57–1.28)	0.450	0.59 (0.38–0.92)	**0.021**
<0 cm	0.30 (0.12–0.78)	**0.013**	0.20 (0.07–0.54)	**0.001**
Treatment and response				
Direct surgery	1.00 (Reference)		1.00 (Reference)	
nCRT (TRG 0–1)	0.68 (0.42–1.12)	0.127	0.62 (0.34–1.10)	0.102
nCRT (TRG 2)	1.50 (0.96–2.35)	**0.070**	1.27 (0.76–2.12)	0.370
nCRT (TRG 3)	1.79 (0.83–3.89)	0.140	1.83 (0.82–4.10)	0.139
Differentiation (poor vs well/moderate)	1.49 (0.76–2.94)	0.250		
Tumor deposit (positive vs negative)	0.69 (0.32–1.47)	0.334		
Lymphovascular invasion (positive vs negative)	1.73 (1.09–2.74)	**0.021**	1.72 (0.96–3.09)	0.070
Perineural invasion (positive vs negative)	1.88 (1.14–3.11)	**0.013**	1.24 (0.69–2.22)	0.473
Postoperative therapy (yes vs no)	0.99 (0.66–1.49)	0.959		

Statistically significant *P*-values with a level of significance set at <0.05 are highlighted in bold. aPR, anterior peritoneal reflection; ASA, American Society of Anesthesiologists; BMI, body mass index; CI, confidence interval; EMVI, extramural vascular invasion; HR, hazard ratio; MRF, mesorectal fascia; mrLLN, magnetic resonance imaging-assessed lateral lymph node; mrN, magnetic resonance imaging-assessed N stage; mrT, magnetic resonance imaging-assessed T stage.

## Discussion

This study identified distinct prognostic differences between upper and middle-to-low rectal cancers, with the classification being defined by the aPR. Notably, nCRT significantly improved DFS exclusively in patients with tumors straddling or located below the aPR and the tumors demonstrated a favorable treatment response (TRG 0–1). Furthermore, only the aPR-defined classification demonstrated significant differences in LLN detection on MRI and the incidence of postoperative pulmonary metastasis across low, middle, and upper rectal cancer subgroups.

Middle-to-low rectal cancers pose significant challenges in preserving anal structure and function and are associated with high rates of positive surgical margins and local recurrence. These factors underscore the rationale behind recommending preoperative therapy for middle-to-low LARC and prioritizing surgical treatment for upper rectal cancers, as reflected in clinical guidelines worldwide. The criteria for selecting patients for nCRT primarily originates from clinical trials. Subgroup analysis from the MRC CR07 study indicated that for tumors located 10–15 cm from the anal verge (measured by rigid sigmoidoscopy), nCRT did not significantly reduce local recurrence rates compared with direct surgery (1.2% vs 6.2%, *P *= 0.19) [17]; similar conclusions were obtained in the Swedish Rectal Cancer Trial [18] and the Dutch TME Trial [19]. Informed by these outcomes, the European Society for Medical Oncology (ESMO) guideline sets the boundary for middle-to-low and upper rectal cancer at 10 cm from the anal verge (measured by rigid sigmoidoscopy), and recommends neoadjuvant therapy for tumors at or below 12 cm from the anal verge [3]. The National Comprehensive Cancer Network (NCCN) guideline uses the aPR as the demarcation for middle-to-low and upper rectal cancer but does not specify a precise threshold for nCRT indications [5]. The CSCO guideline defines middle-to-low rectal cancer as tumors <10 cm from the anal verge (measured by MRI), with tumors above this level being managed similarly to colon cancer [4].

In this context, we contend that any instrument or metric employed to define tumor location must possess both robust theoretical and practical significance. Relying on a fixed distance from the anal verge as the benchmark for nCRT may introduce potential measurement inaccuracies that could significantly impact treatment strategies [20]. Moreover, this approach may overlook anatomical variations, including sex, weight, and pelvic morphology, causing inconsistencies across national guidelines [21]. An international, expert-based Delphi consensus identified MRI as the preferred imaging modality and defined the upper rectum as the segment of the bowel extending from the sigmoid take-off to the PR [22]. The PR, as an anatomical structure, exhibits considerable individual variation. Previous researches have extensively explored the aPR due to its clear visibility. Cadaveric studies indicated that the aPR height ranges from 7.0 to 9.0 cm in males and 5.0 to 7.5 cm in females, aligning with the second Houston valve [23]. Intraoperative rigid sigmoidoscopy studies found the average aPR height to be 9.7 cm in males and 9.0 cm in females [24]. Recent MRI-based study reported aPR heights of 9.62 ± 1.47 cm in males and 9.57 ± 0.68 cm in females, with MRI achieving approximately 90% accuracy in aPR identification [25]. Factors such as sex, height, weight, and pelvic morphology are associated with aPR height, though considerable heterogeneity exists due to differences in measurement methods and study designs. Data from our center (432 cases) demonstrated an average aPR height of 98.7 ± 14.4 mm (range, 63.6–154 mm) in Chinese patients with rectal cancer, closely relating to weight and pelvic morphology, as revealed in the multivariate analysis, suggesting potential ethnic differences [8]. While the average aPR height approximates 10 cm, patient subgroups defined by different criteria exhibit heterogeneity in prognosis and treatment efficacy. Gao *et al*. [6] demonstrated that patients with rectal cancers located above the aPR have significantly longer local recurrence-free survival than those with cancers straddling or below the aPR. Postoperative radiotherapy improved local recurrence-free survival only in tumors straddling or below the aPR, but provided no additional benefit for tumors above it. Our findings showed significant DFS differences between aPR-defined subgroups were absent when fixed distance-defined stratification was used. Additionally, the prognostic benefit of nCRT depended on tumor location and response: only middle-to-low rectal cancers (straddling or below the aPR) with a favorable nCRT response showed DFS improvements. Rectal cancers above the aPR are intraperitoneal tumors, for which high-quality surgery may ensure R0 resection and result in satisfactory oncological outcomes, with minimal additional benefit from nCRT. The response of tumors to nCRT is recognized as an independent predictor of prognosis [26, 27]. Re-examining previous studies, it is noted that there were low proportions of well responders in trials such as CAO/ARO/AIO-94 [28] and EORTC 22921 [29] (1.9% pCR in the experimental group of CAO/ARO/AIO-94, and 15% pCR and near-pCR in the experimental group of EORTC 22921), with inclusion of 10%–20% “upper rectal cancer” patients. These factors may partly account for the lack of observed survival benefit from nCRT in these studies. Thus, in contemporary clinical practice, employing total neoadjuvant therapy [30, 31] or integrating immunotherapy [32, 33] for middle-to-low LARC below the aPR may achieve higher pCR rates and long-term prognostic benefits.

LLNs represent a unique lymphatic drainage area for middle-to-low rectal cancers. Previous studies showed that the presence of lateral lymph nodes on baseline MRI is associated with poor prognosis [34]. The optimal treatment standards remain controversial, underscoring the importance of preoperative identification and risk assessment [35]. The metastatic pattern of LLNs shares similarities with that of tumors at the esophagogastric junction, where tumor classification and treatment strategy are influenced by its relationship to the esophagogastric junction [14]. For example, the extent of esophageal invasion in Siewert type II adenocarcinoma determines the necessity of mediastinal lymphadenectomy [36]. However, to the best of our knowledge, no studies have yet quantitatively defined tumor location using the aPR as a reference to predict LLN metastasis in rectal cancer. This study applied esophagogastric junction adenocarcinoma concepts and used MRI to assess tumor location relative to the aPR, comparing it with the tumor height for predicting LLN metastasis, as prior studies identified the latter as an independent risk factor [37]. Our results demonstrated that using the aPR as a reference for quantitatively defining tumor location was superior. Nevertheless, further investigation involving LLN dissection and pathological examination is required to validate these findings.

Regarding metastatic patterns, pulmonary metastasis occurs more frequently in patients with rectal cancer than in those with colon cancer [38]. Tumor location is a key factor, with studies confirming that pulmonary metastasis risk in rectal cancer rises as the tumor height decreases [39, 40]. This pattern is associated with the venous and lymphatic drainage pathways. The tumor position relative to the aPR is theoretically more accurate for predicting pulmonary metastasis risk. Our study validated this hypothesis, providing new insights into how tumor location affects recurrence patterns in rectal cancer.

This study has several limitations. First, being retrospective, this study inherently has selection bias. For instance, our center predominantly conducts rectal MRI for middle-to-low rectal cancer, resulting in a higher proportion of such cases than that of upper rectal cancer. Second, this single-center study has a limited number of cases. Our center primarily follows the CSCO guideline, using an MRI-measured distance of ≤10 cm from the tumor to the anal verge to select rectal cancer patients for nCRT. This results in fewer cases of tumors located above the aPR receiving nCRT, reducing statistical power in the Cox model subgroup analysis for these cases. However, it does indicate that DFS improvement with nCRT is confined to patients with tumors straddling or below the aPR. Third, heterogeneity in prognosis and therapeutic efficacy due to varying tumor location definitions stems from a minority group with conflicting criteria. Therefore, direct analyses of such populations may be more appropriate and could serve as a sensitivity analysis to further validate our conclusions. However, due to the aforementioned limitations, this study focused only on the overall impact of this contradictory subgroup, which is a compromise.

In conclusion, defining tumor location by the aPR is of guiding value in the decision-making for nCRT in LARC. Tumors straddling or below the aPR have significantly shorter DFS than those above the aPR, and may derive DFS benefits from nCRT. Using the aPR, rather than the anal verge, as a reference point enhances clinical applicability by better predicting LLN metastasis and patterns of distant organ metastasis. In summary, the aPR provides potentially decisive guidance in diagnosing and treating rectal tumors, which requires further validation in multi-center, prospective studies.

## Authors’ contributions

All authors read and approved the final manuscript. H.Z. conceived, designed and conducted the study, collected, analyzed and interpreted the data, and drafted the manuscript. G.L., B.W., H.Q., J.L., X.S., B.N., L.X., G.Z., Z.S., and K.L. contributed to data collection and manuscript preparation. Y.X. conceived, designed and conducted the study, critically reviewed the intellectual content of the manuscript, secured funding, provided administrative, technical, or material support, and contributed guidance and supportive input.
